# Undersampled MR Image Reconstruction with Data-Driven Tight Frame

**DOI:** 10.1155/2015/424087

**Published:** 2015-06-24

**Authors:** Jianbo Liu, Shanshan Wang, Xi Peng, Dong Liang

**Affiliations:** ^1^Paul C. Lauterbur Research Centre for Biomedical Imaging, Shenzhen Institutes of Advanced Technology, Chinese Academy of Sciences, Shenzhen 518055, China; ^2^School of Information Technologies, The University of Sydney, Sydney, NSW 2006, Australia

## Abstract

Undersampled magnetic resonance image reconstruction employing sparsity regularization has fascinated many researchers in recent years under the support of compressed sensing theory. Nevertheless, most existing sparsity-regularized reconstruction methods either lack adaptability to capture the structure information or suffer from high computational load. With the aim of further improving image reconstruction accuracy without introducing too much computation, this paper proposes a data-driven tight frame magnetic image reconstruction (DDTF-MRI) method. By taking advantage of the efficiency and effectiveness of data-driven tight frame, DDTF-MRI trains an adaptive tight frame to sparsify the to-be-reconstructed MR image. Furthermore, a two-level Bregman iteration algorithm has been developed to solve the proposed model. The proposed method has been compared to two state-of-the-art methods on four datasets and encouraging performances have been achieved by DDTF-MRI.

## 1. Introduction

Magnetic Resonance Imaging (MRI) is one of the major diagnostic imaging modalities with noninvasive and nonionizing radiation nature. However, relatively low imaging speed limits its wide application in clinic [1]. To accelerate MRI, one popular way is to reduce the number of acquired data [2]. With *f* ∈ *ℂ* ^*m*^ and *x* ∈ *ℂ* ^*n*^, respectively, denoting the *K*-space measurement and the original image, the imaging model could be described as follows:

(1) $$\begin{array}{c} f=PFx+\epsilon =F_{p}x+\epsilon , \end{array}$$

where *ϵ* is the complex additive white Gaussian noise with standard deviation *σ*, *P* is the undersampling operator, *F* is the Fourier operator, and *F*_*p*_ = *PF*.

Recovering *x* from the undersampled measurement *f* is an ill-posed inverse problem. To address this ill-posed nature, it is necessary to utilize some prior knowledge to regularize the MR image so as to make up the missing information. With the popularity of compressed sensing (CS) theory [3, 4], the sparsity-promoting regularization for MR image reconstruction has attracted many researchers [2, 5, 6]. Specifically, the CS theory has shown that if an image has a sparse representation under certain transform, we can precisely restore the original image from its partial measurements under the RIP condition [3, 4]. With such a transform, the MR image reconstruction from its undersampled *K*-space data can be achieved by nonlinear algorithms, like *ℓ*_1_ minimization or orthogonal match pursuit (OMP) algorithm [2, 5, 6].

The commonly used transforms in MR image reconstruction include total variation (TV) and wavelet transform, both of which can be regarded as tight frames [7–13]. As the tight frame satisfies the perfect reconstruction property, which ensures that the given signal can be perfectly represented by its canonical representation, it has been leveraged in diverse inverse problems [7, 11, 12]. However, TV prior assumes that the image consists of piecewise constant areas which may not be valid in many real MR images. When the measurements are highly undersampled, compressed sensing based MRI using TV regularization (CSMRI-TV) could lead to severe blocky artifacts [2, 14]. To improve the image quality, Liang et al. [15] applied the nonlocal total variation (NLTV) regularization in parallel MR imaging by replacing the gradient functional in conventional TV with a weighted nonlocal gradient function. However, although NLTV reduces the blocky effects, it is still based on fixed transform, which does not adapt to the target image. Furthermore, other analytically designed X-lets [8–10, 16], such as wavelets and shearlets, also suffer from their intrinsic deficiencies [17], such as the dependency between parent and child wavelet coefficients and the lack of adaptability. Qu et al. [18] introduced the patch-based directional wavelet (PBDW) in MR image reconstruction by exploiting the geometric direction of image patches, which has shown encouraging performances on edge preservation and noise removal. Nevertheless, PBDW is still a simplified form of bandlets and the adaptability can still be explored [19].

Since the fixed tight frame/transform might not be universally optimal for all images, the data-driven tight frame/transform has been developed [5, 6, 20, 21] to adaptively capture the structure information. One most popular direction is the incorporation of the dictionary learning (DL) into MRI [6, 21, 22], among which Ravishankar and Bresler proposed a benchmark work named DLMRI [5] with outstanding reconstruction results achieved. However, dictionary learning based optimization is a large-scale and highly nonconvex problem, which requires high computational complexity [6, 20]. More recently, Cai et al. [20] presented a scheme to learn a data-driven tight frame from the measurements and applied it to solve the image denoising problem. As the data-driven tight frame satisfies the perfect reconstruction property, it is very efficient to obtain the results with less artifacts. Therefore, Wang et al. [23] tried to incorporate data-driven tight frame into dynamic MRI. However, the proposed method relies on a perfect reference image, which is quite hard to be obtained from its undersampled *K*-space data, and the data-driven tight frame is learnt from the reference image instead of the target image.

Based on these observations and motivated by the efficiency and effectiveness of the data-driven tight frame (DDTF), in this work, we propose an undersampled MR imaging method by incorporating DDTF into the reconstruction model with the aim of further improving the image reconstruction accuracy without introducing too much computation. Specifically, a tight frame has been adaptively trained for each to-be-reconstructed MR image. To solve the proposed model, a two-level Bregman iteration algorithm has been developed. We name the proposed approach as DDTF-MRI and compare it to two state-of-the-art MR image reconstruction algorithms, including CSMRI-TV and DLMRI, on one simulated MR image and three in vivo datasets under different undersampling scenarios. The experiments have shown encouraging performances of the proposed method.

The rest of the paper is organized as follows.  Section 2 presents the preliminary and previous work. Our proposed method DDTF-MRI is illustrated in Section 3. The experimental results are provided in Section 4 demonstrating that the proposed algorithm has potential to improve the MR image reconstruction from its undersampled *K*-space data. Finally, conclusions are given in Section 5.

## 2. Preliminary and Previous Work

### 2.1. Tight Frame

A system {*x*_*i*_} ⊂ *L*_2_(*ℂ*) is a tight frame [7, 24] of *L*_2_(*ℂ*) if

(2) $$\begin{array}{c} g=\underset{i}{\sum }\langle \;g,x_{i}\;\rangle x_{i},\;\forall g\in L_{2}\left(C\right), \end{array}$$

where 〈·, ·〉 is the inner product operator and the space *L*_2_(*ℂ*) represents the set of all the functions *f*(*θ*) satisfying ‖*f*‖_*L*_2_(*ℂ*)_≔(∫_*ℂ*_ | *f*(*θ*) | *dθ*)^1/2^. For a given tight frame, we can define the corresponding analysis operator *W* as

(3) $$\begin{array}{c} W:g\in L_{2}\left(\;C\;\right)⟶\left\{\;\langle \;x,x_{i}\;\rangle \;\right\}\in l_{2}\left(\;N\;\right) \end{array}$$

and the synthesis operator *W* ^*T*^ as

(4) $$\begin{array}{c} W^{T}:\left\{\;c_{i}\;\right\}\in l_{2}\left(\;N\;\right)⟶\underset{n}{\sum }c_{i}x_{i}\in L_{2}\left(\;C\;\right). \end{array}$$

The system is a tight frame if and only if *W* ^*T*^ *W* = *I*, where *I* is the identity operator. Moreover, a tight frame can be generated from a filter bank satisfying the Unitary Extension Principle (UEP) condition [7]. Let {*a*_*i*_}_*i*=1_^*i*=*r*^2^^ denote the set of 2D filters and the size of *a*_*i*_ is *r* × *r* and then the synthesis operator *W* ^*T*^ ∈ *ℂ* ^*n*×*nr*^2^^ and the analysis operator *W* ∈ *ℂ* ^*nr*^2^×*n*^ are defined as

(5) WT=Sa1,Sa2,…,Sar2,W=Sa1T,Sa2T,…,Sar2TT,

where *S*_*a*_*i*__ ∈ *ℂ* ^*n*×*n*^ denotes the convolution matrix associated with the filter *a*_*i*_, which is a block-wise Toeplitz matrix under Neumann boundary conditions [7, 24].

### 2.2. Sparsity-Regularized MR Image Reconstruction

This subsection briefly introduces two representative sparsity-regularized MR image reconstruction methods, namely, CSMRI and DLMRI. CSMRI is a classical approach exploiting sparsity via fixed transform and its mathematical model can be defined as follows:

(6) min⁡x Wx1,s.t. f−Fpx2≤σ2,

where *W* is the analysis operator of its corresponding frame system, that is, wavelet or total variation.

DLMRI proposed by Ravishankar and Bresler [5], on the other hand, employs the *K*-singular value decomposition (*K*-SVD) algorithm [21] to learn an adaptive dictionary to sparsely represent the patches extracted from the target image. The DLMRI model can be written as

(7) min⁡x,D,Γ ∑iRix−Dαi22+λ∑iαi0,s.t. f−Fpx2≤σ2,

where *D* represents the dictionary, *R*_*i*_ denotes the operator that extracts the *i*th patch from the image *x*, and Γ denotes the set of all sparse coefficients {*α*_*i*_}.

## 3. The Proposed DDTF-MRI Method

### 3.1. Image Reconstruction Model

To enhance the sparsity in the frame domain, the proposed DDTF-MRI method is devoted to learning an adaptive data-driven tight frame to effectively sparsify the target image. The proposed model can be described as

(8) min⁡x,W∈Λ Wx1,s.t. f−Fpx2≤σ2,

where Λ = {*W*∣*W* ^*T*^ *W* = *I*} and the tight frame *W* is constructed by the corresponding 2D filters {*a*_*i*_}_*i*=1_^*i*=*r*^2^^ in the manner of (5).

### 3.2. The Proposed Algorithm

A two-level Bregman iteration algorithm has been developed to attack the target model.

#### 3.2.1. The Outer Bregman Iteration

In the outer-level Bregman iteration, we try to solve the following problem [6, 25, 26]:

(9) xk+1,Wk+1=argminx,W∈Λ⁡Wx1+μ2Fpx−f+ck22,ck+1=ck+δcFpxk+1−f,

where *μ* > 0 and *δ*_*c*_ ∈ (0,2) [26].

#### 3.2.2. The Inner Bregman Iteration

With introduction of an assistant variable, *v* = *Wx*, we can obtain the constrained version of the first subproblem in (9):

(10) min⁡x,W∈Λ,v v1+μ2Fpx−f+ck22,s.t. v=Wx.

Employing the Bregman iteration technique again under the assistance of a dual variable, *b*, in the inner-level Bregman iteration, we target solving

(11) xk+1=argminx⁡μ2Fpx−f+ck22+λ2Wx−vk+bk22,vk+1,Wk+1=argminv,W∈Λ⁡v1+λ2Wxk+1−v+bk22,bk+1=bk+δbWk+1xk+1−vk+1,

where *λ* > 0 and 0 < *δ*_*b*_ ≤ 1 [26].

#### 3.2.3. Subproblems of (11)

For the *x*-subproblem in (11), as *W* ^*T*^ *W* = *I*, its least squares solution can be obtained as

(12) $$\begin{array}{c} \left(\;\mu F_{p}^{T}F_{p}+\lambda I\;\right)x^{k+1}=\mu F_{p}^{T}\left(\;f-c^{k}\;\right)+\lambda W^{T}\left(\;v^{k}-b^{k}\;\right). \end{array}$$

Multiplying both sides of (12) by *F* and letting *S*_1_ = *f* − *c* ^*k*^ and *S*_2_ = *F*[*W* ^*T*^(*v* ^*k*^ − *b* ^*k*^)], we obtain the interpolation formulation in the frequency domain:

(13) Fxk+1kx,ky=S2kx,ky,kx,ky∉Ω,μS1kx,ky+λS2kx,kyμ+λ,kx,ky∈Ω,

where *Ω* stands for the subset of *K*-space that has been sampled. The image can then be updated via the inverse Fourier transform.

Next, we apply the alternating-minimization strategy to solve the {*v*, *W*}-subproblems:

(14) $$\begin{array}{c} \underset{v,W\in \Lambda }{\min}{\left\|\;v\;\right\|}_{1}+\frac{\lambda }{2}{\left\|\;Wx^{k+1}-v+b^{k}\;\right\|}_{2}^{2}. \end{array}$$

With *W* temporarily fixed, the *v*-subproblem becomes

(15) $$\begin{array}{c} v^{k+1/2}=\underset{v}{\mathrm{argmin}}{\left\|\;v\;\right\|}_{1}+\frac{\lambda }{2}{\left\|\;Wx^{k+1}-v+b^{k}\;\right\|}_{2}^{2}. \end{array}$$

Following the iterative shrinkage/thresholding algorithm (ISTA) [27], it yields

(16) $$\begin{array}{c} v^{k+1/2}=\mathrm{shrink}\left(\;Wx^{k+1}+b^{k},\frac{1}{\lambda }\;\right), \end{array}$$

where shrink(*x*, *a*) = sign⁡(*x*)max⁡{0, |*x* | − *a*}. Now let *v* be kept fixed; the *W*-subproblem turns to be

(17) $$\begin{array}{c} W^{k+1}=\underset{W\in \Lambda }{\mathrm{argmin}}{\left\|\;Wx^{k+1}-v^{k+1/2}+b^{k}\;\right\|}_{2}^{2}. \end{array}$$

Instead of directly optimizing *W*, we apply the technique of [20] to solve this subproblem by using SVD to obtain the corresponding filters {*a*_*i*_}.

Furthermore, for a better restoration result, we also update the variable *v* in the updated tight frame domain

(18) $$\begin{array}{c} v^{k+1}=\underset{v}{\mathrm{argmin}}{\left\|\;v\;\right\|}_{1}+\frac{\lambda }{2}{\left\|\;W^{k+1}x^{k+1}-v+b^{k}\;\right\|}_{2}^{2} \end{array}$$

and obtain

(19) $$\begin{array}{c} v^{k+1}=\mathrm{shrink}\left(\;W^{k+1}x^{k+1}+b^{k},\frac{1}{\lambda }\;\right). \end{array}$$

To help the readers grasp the overall picture better, we summarize the entire process in Algorithm 1.

## 4. Results and Discussion

### 4.1. Experimental Setup

We evaluated the proposed DDTF-MRI on four datasets and compared it to two state-of-the-art methods, including CSMRI-TV (the CSMRI-TV code is available in http://www.eecs.berkeley.edu/~mlustig/CS.html) and DLMRI (the DLMRI code is available in http://www.ifp.illinois.edu/~yoram/DLMRI-Lab/DLMRI.html) [5]. The four datasets consist of one simulated data and three in vivo datasets. The simulated data is from the paper in [5]. For the in vivo data, informed consent was obtained from the volunteer in accordance with the institutional review board policy. The first in vivo data is a sagittal brain collected on a GE 3T scanner (GE Healthcare, Waukesha, WI, USA) with 32-channel head coil and 3D T1-weighted spoiled gradient echo sequence: TE = minimum full, TR = 7.5 ms, FOV = 24 × 24 cm, matrix = 256 × 256, and slice thickness = 1.7 mm. The heart dataset was acquired on a 1.5 T Philips scanner using the steady-state free precession (SSFP) sequence with a flip angle of 50 degree and TR = 3.45 msec. The field of view (FOV) was 345 mm × 270 mm and the slice thickness was 10 mm. Retrospective cardiac gating was used with a heart rate of 66 bpm. The third in vivo data was acquired on a 3 T commercial scanner (GE Healthcare, Waukesha, WI, USA) and eight-channel head coil (Invivo, Gainesville, FL, USA) with a 2D T1-weighted spin-echo protocol (axial plane, TE/TR = 11/700 ms, 22 cm FOV, 10 slices, matrix size = 256 × 256). The adaptive combination method [28] is applied to integrate the multichannel data into a single-channel complex-valued image before we apply the three methods. Then the full *K*-space data corresponding to the single channel image is retrospectively undersampled under different sampling schemes.

**Figure 1:**
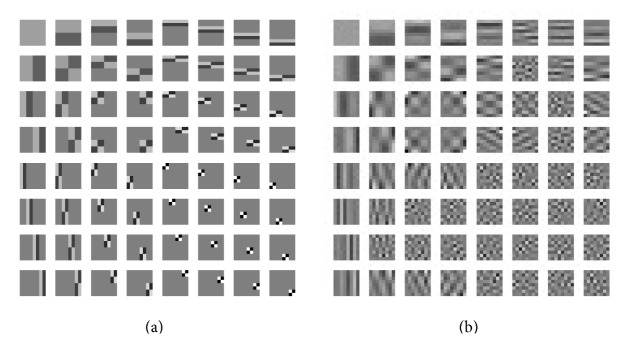
(a) Original filters. (b) The corresponding learnt filters in the experiment of Figure 2.

As shown in Figure 1(a), we employed the 3-level shift-invariant Haar wavelet filters (the size of each filter is 8 × 8) as the initialization of the tight frame in DDTF-MRI. As for the parameter settings, both CSMRI-TV and DLMRI were implemented with their default settings. For DDTF-MRI, we set *M* = 3, *δ*_*b*_ = 1, *δ*_*c*_ = 1, *μ* = 10, and *λ* = 10. The outer-level iteration of DDTF-MRI continues until *k* > 25. Furthermore, we used both the peak signal-to-noise ratio (PSNR) (the PSNR is defined as PSNR = 20log_10_255/RMSE, where the RMSE is the root mean error estimated between the ground truth and the reconstructed image), high-frequency error norm (HFEN) [5], and structural similarity (SSIM) index [29] for a quantitative comparison of recovered results.

### 4.2. Test on the Simulated Data

**Figure 2:**
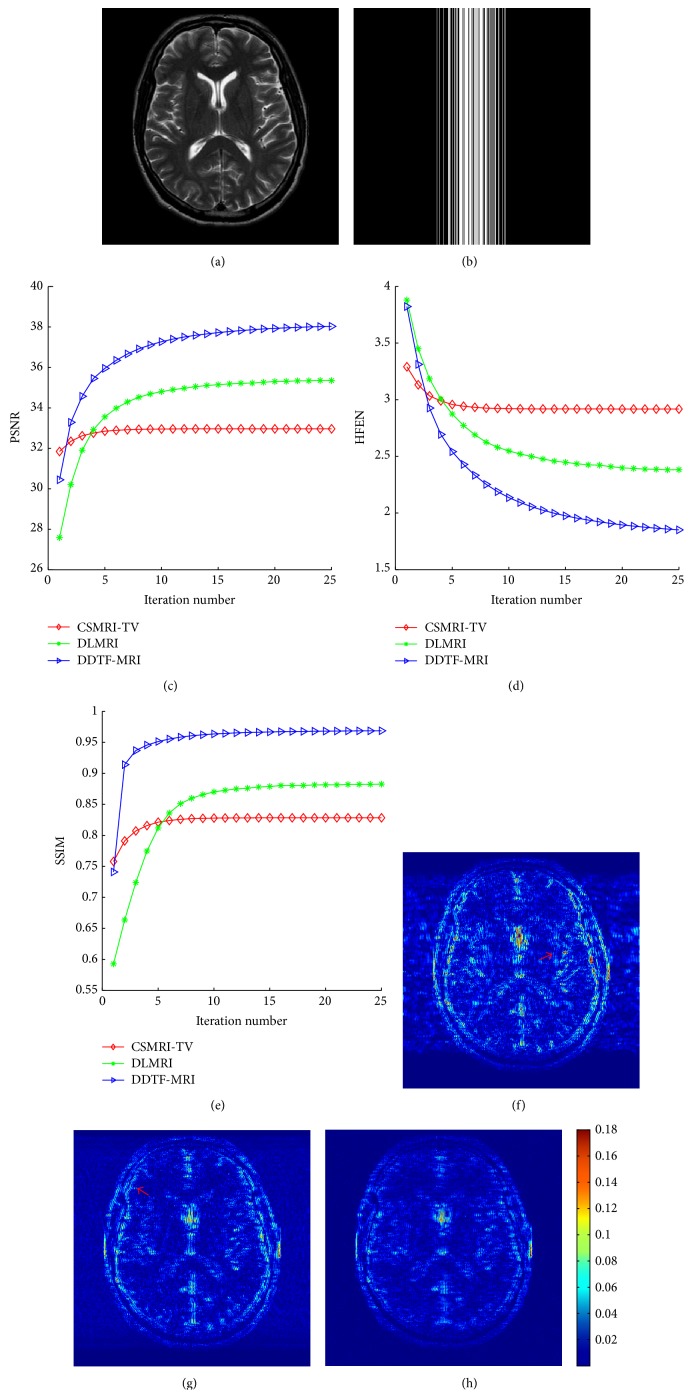
(a) Original image. (b) Cartesian sampling mask with accelerating factor *R* = 6.7. (c), (d), and (e) are the PSNR, HFEN, and SSIM values versus the number of iterations. (f), (g), and (h) are reconstruction error magnitudes for CSMRI-TV, DLMRI, and DDTF-MRI, respectively.

We firstly applied each algorithm to reconstruct the simulated T2-weighted image under the 1D Cartesian sampling schemes with accelerating factor *R* = 6.7 (sampling ratio 15%). The filters learned by DDTF-MRI are shown in Figure 1(b). Compared with the initialization filter presented in Figure 1(a), we can see that the learnt filters have captured more directional information. Figures 2(a) and 2(b), respectively, present the original image of size 512 × 512 and the sampling mask. The evolution of the PSNR, HFEN, and SSIM values over iteration number is plotted in Figures 2(c), 2(d), and 2(e), which indicates that our proposed method is superior to the other two algorithms. The final PSNR values obtained by CSMRI-TV, DLMRI, and DDTF-MRI are, respectively, 32.97 dB, 35.34 dB, and 38.03 dB. Figures 2(f), 2(g), and 2(h) provide the absolute reconstruction differences between the original image and the results obtained by the three methods, from which we can see the proposed method produces less estimation error. Moreover, a region-of-interest (ROI) analysis was performed by calculating means and standard deviations in selected ROIs.  Table 1 shows the numerical result calculated from three selected ROIs in this experiment. The numbers of pixels in ROI 1, ROI 2, and ROI 3 are 1944, 1248, and 1944, respectively. From Table 1, we can find the values of our reconstruction result are closest to the values of reference image, which illustrates that our method can give a more precise reconstruction result.

### 4.3. Test on In Vivo Datasets

**Figure 3:**
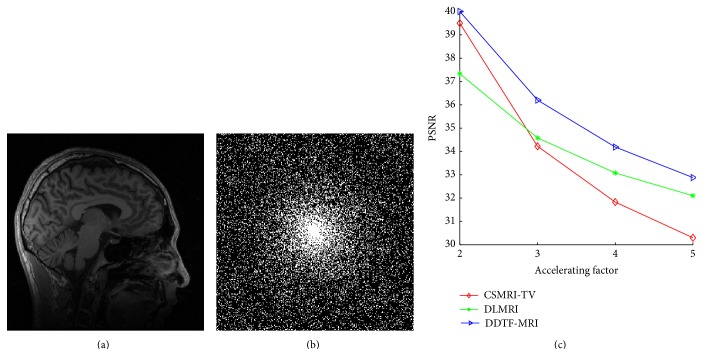
(a) Original image. (b) Random sampling mask. (c) PSNR values versus accelerating factors.

**Figure 4:**
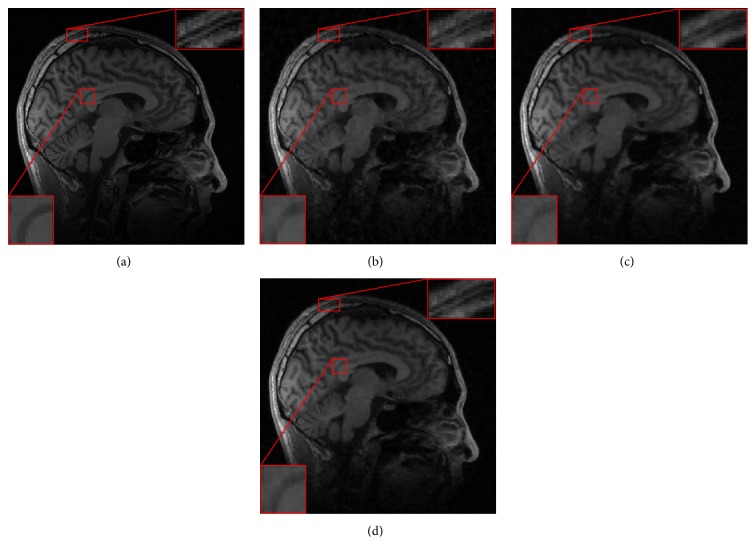
(a) Reference. Reconstructed images from the random sampling mask with accelerating factor *R* = 3 by (b) CSMRI-TV, (c) DLMRI, and (d) our proposed DDTF-MRI, respectively.

We then employed these three methods to reconstruct the sagittal brain and test their sensitivity to the acceleration factors.  Figure 3(c) plots the PSNR values versus different accelerating factors under the random sampling trajectory. It shows that DDTF-MRI performs better than the other two methods at all the acceleration factors. For a visual comparison, Figure 4 provides the reconstructed results at accelerating factor 3. For a close-up comparison, we have enlarged two edge parts, from which we can see DDTF-MRI provides clearer details.

**Figure 5:**
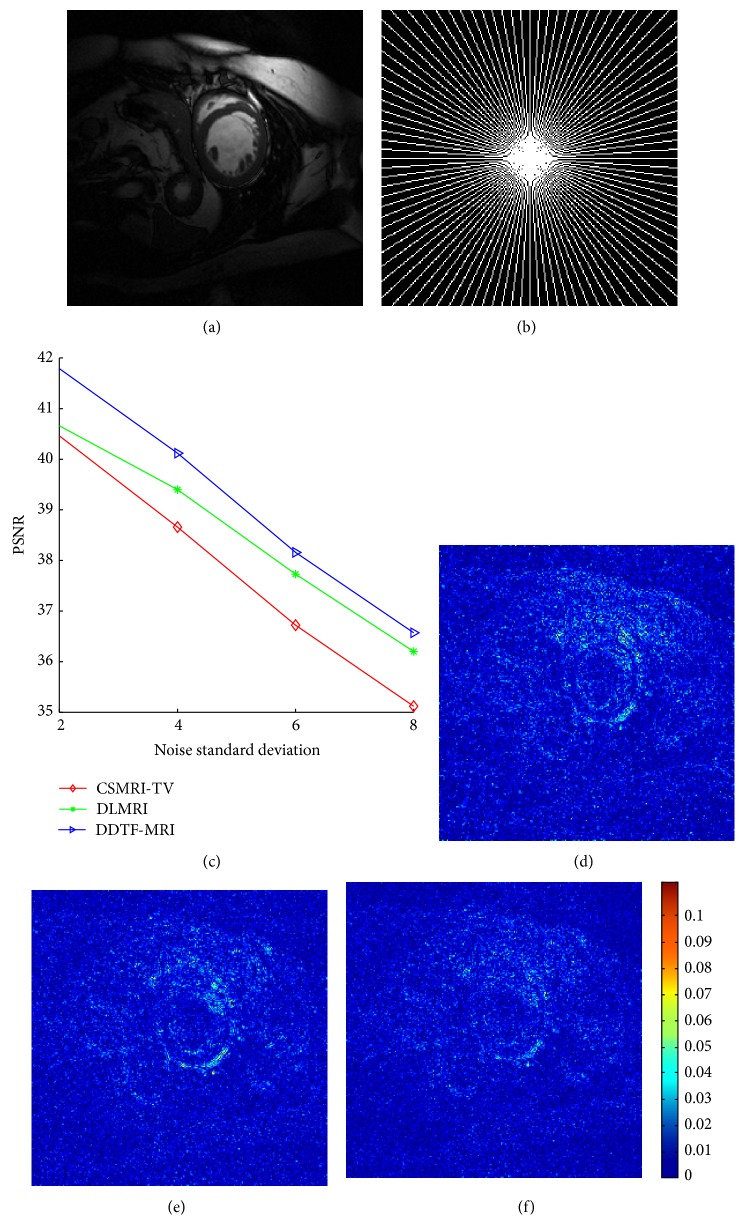
(a) Original image. (b) Radial sampling mask. (c) PSNR versus noise standard deviation. The error magnitudes of reconstructed results from the 3-fold radial sampling mask with noise standard deviation 4 by (d) CSMRI-TV, (e) DLMRI, and (f) our proposed DDTF, respectively.

To investigate the sensitivity of various methods to noise, CSMRI-TV, DLMRI, and DDTF-MRI were applied to reconstruct the heart image under pseudoradial sampling scheme with accelerating factor *R* = 3.  Figure 5(c) provides the PSNR values of the MR images reconstructed by CSMRI-TV (red curves), DLMRI (green curves), and DDTF-MRI (blue curves) versus diverse noise levels (*σ* = 2, 4,6, 8). We can see that our proposed DDTF-MRI exhibits the best performance for these noise levels. Figures 5(d), 5(e), and 5(f) show the reconstructed error magnitudes from the 3-fold radial undersampled data with noise standard deviation as 4. We can find that our method can preserve more detail and recover the structures more precisely.

**Figure 6:**
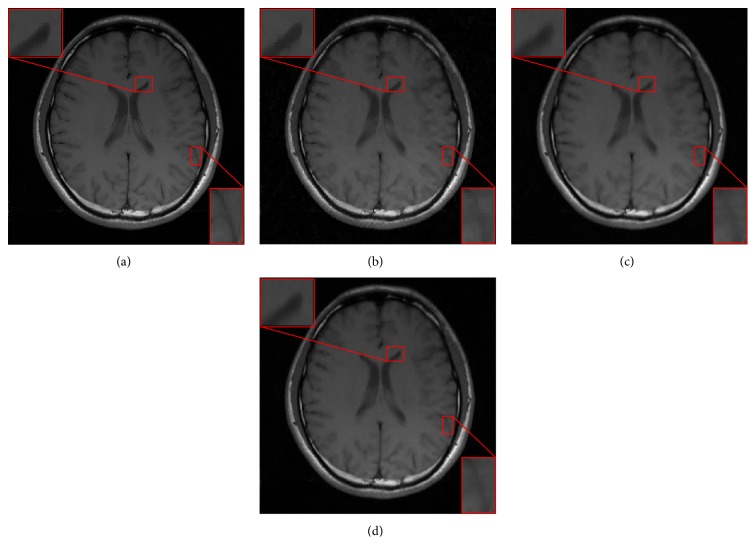
(a) Reference. Reconstructed images from the radial sampling mask with accelerating factor *R* = 4 by (b) CSMRI-TV, (c) DLMRI, and (d) our proposed DDTF-MRI, respectively.

In Figure 6, we evaluated the proposed method with another in vivo brain data, which contains more fine-detailed structures, using the radial sampling trajectory. The reconstructed results using CSMRI-TV, DLMRI, and DDTF-MRI with a higher acceleration factor *R* = 4 are displayed in Figures 6(b), 6(c), and 6(d), respectively. The zoom-in results are also provided in Figure 6. Compared to the reference image shown in Figure 6(a), it can be observed that the result obtained by CSMRI-TV suffers from blocky artifacts. Meanwhile, the reconstructed image obtained by DDTF-MRI is clearer and sharper than those reconstructed by CSMRI-TV and DLMRI. This reveals that our proposed method can provide a more accurate recovered image.

### 4.4. Computation Time

In our experiments, all algorithms were implemented in MATLAB and performed on a laptop equipped with Intel 2.60 GHz CPU and 4 GB RAM.  Table 2 illustrates the cost time for these experiments. These computational times were obtained by averaging 10 times for each experiment. The size of the simulated image (Figure 2(a)) is 512 × 512 and the size of complex-valued images (Figures 4(a), 5(a), and 6(a)) is 256 × 256. As CSMRI-TV is based on fixed transform, it is the fastest. However, this efficiency comes at the cost of accuracy. DLMRI takes the longest time in all cases. On the other hand, DDTF-MRI has achieved the best reconstruction result without taking the longest reconstruction time, which is due to three aspects: (1)  *ℓ*_1_ norm is used in DDTF-MRI instead of *ℓ*_0_ norm. While *ℓ*_0_ norm minimization is a NP-hard problem, *ℓ*_1_ norm minimization is convex and can be solved with much less time while promoting sparsity; (2) the tight frame used in this paper ensures that the given signal can be perfectly represented by its canonical expansion as *W* ^*T*^ *W* = *I*; therefore it can be efficiently implemented; (3) two-level Bregman iteration technique is used in the proposed method, which can further promote fast and accurate MR image reconstruction. So the proposed method has improved the accuracy without sacrificing too much efficiency.

## 5. Conclusions

A DDTF-MRI method has been proposed in this paper to effectively reconstruct MR image from undersampled *K*-space data. DDTF-MRI trains an adaptive tight frame for each to-be-reconstructed image. Furthermore, a two-level Bregman iteration algorithm has been developed to solve the proposed model. Results on both simulated and in vivo datasets demonstrate the superior performance of DDTF-MRI over the other two state-of-the-art MR image reconstruction methods, including CSMRI-TV and DLMRI, in artifacts suppression and edge preservation. Although DDTF-MRI is slower than the fixed transform based method CSMRI-TV, its accuracy is much higher than CSMRI-TV. Furthermore, as an adaptive training method, DDTF-MRI is much faster than the typical training method, DLMRI. This indicates that our method can improve the reconstruction accuracy without introducing too much computation load. In the future, we may further optimize the implementation and consider sparser representations.

## Figures and Tables

**Algorithm 1:**
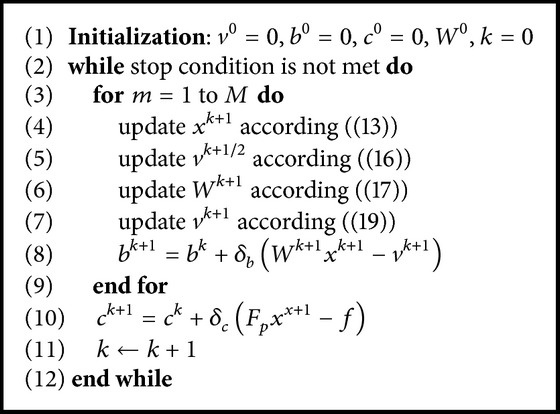
DDTF-MRI.

**Table 1:** ROI analysis of mean and standard deviation (std).

Image	Different ROIs		
	ROI 1	ROI 2	ROI 3
	Mean ± Std	Mean ± Std	Mean ± Std
Reference	35.34 ± 5.55	29.72 ± 2.97	39.08 ± 5.87
CSMRI-TV	35.47 ± 7.31	29.69 ± 3.70	39.01 ± 7.89
DLMRI	35.14 ± 4.77	29.40 ± 2.04	38.83 ± 5.19
DDTF-MRI	35.33 ± 5.35	29.75 ± 2.67	39.09 ± 5.86

**Table 2:** Computational time (s) of different algorithms.

Image	Different algorithms		
	CSMRI-TV	DLMRI	DDTF-MRI
Figure 2(a)	147.3	1600.6	310.2
Figure 4(a)	30.9	2609.2	314.5
Figure 5(a)	37.1	2484.3	315.3
Figure 6(a)	31.5	2503.6	313.7
